# Flipped classroom instructional approach in undergraduate medical education

**DOI:** 10.12669/pjms.336.13699

**Published:** 2017

**Authors:** Syeda Sadia Fatima, Fazal Manzoor Arain, Syed Ather Enam

**Affiliations:** 1 Syeda Sadia Fatima, MBBS, M.Phil, PhD. Department of Biological and Biomedical Sciences, The Aga Khan University, Karachi, Pakistan; 2 Fazal Manzoor Arain, MBBS, PhD. Department of Biological and Biomedical Sciences, The Aga Khan University, Karachi, Pakistan; 3 Syed Ather Enam, MD, PhD, FRCS, FRCS, FACS. Department of Surgery, The Aga Khan University, Karachi, Pakistan

**Keywords:** E-Learning, Flipped classroom, Medical Education, Medical students

## Abstract

**Objective:**

In this study we implemented the “flipped classroom” model to enhance active learning in medical students taking neurosciences module at Aga Khan University, Karachi.

**Methods:**

Ninety eight undergraduate medical students participated in this study. The study was conducted from January till March 2017. Study material was provided to students in form of video lecture and reading material for the non-face to face sitting, while face to face time was spent on activities such as case solving, group discussions, and quizzes to consolidate learning under the supervision of faculty. To ensure deeper learning, we used pre- and post-class quizzes, work sheets and blog posts for each session. Student feedback was recorded via a likert scale survey.

**Results:**

Eighty four percent students gave positive responses towards utility of flipped classroom in terms of being highly interactive, thought provoking and activity lead learning. Seventy five percent of the class completed the pre-session preparation. Students reported that their queries and misconceptions were cleared in a much better way in the face-to-face session as compared to the traditional setting (4.09 ±1.04).

**Conclusion:**

Flipped classroom(FCR) teaching and learning pedagogy is an effective way of enhancing student engagement and active learning. Thus, this pedagogy can be used as an effective tool in medical schools.

## INTRODUCTION

Students frequently believe they fully understand a topic while it is being covered in class, but actually they do not.^1^ Furthermore, the problems surrounding effective learning are compounded by the fact that every student is unique and learns in different styles. To maximize students learning, teachers need to be aware of how the students learn, and adjust their teaching strategy to fit the student needs.^2^

The notion of “flipped classroom” (FCR) is an emerging concept in education which is gaining much popularity.^3^ The millennial students are quiet adaptive in using technology and prefer this mode for teaching and learning.^4^ They are comfortable while learning in social situations. Therefore it is imperative to seek ideas that utilize e-learning technologies as potent promoters for active self-directed deeper learning in our education systems.^5^ Teachers who use the ‘flip classroom approach’ reverse the role of school work and homework; they achieve this by either recording their lectures or using already available video lectures from the internet. Students watch these lectures before coming to class. They then in turn get a chance to discuss the topic in the classroom in their preferred social learning style with the guide of the facilitator.

Limited data is available on the effectiveness of the flipped classroom to determine if students are engaged more in a flipped classroom environment, especially for medical school demographics.^6^,^7^ Investigating the benefits, shortcomings, student perceptions, and later the academic results of this teaching method is important to medical education on several levels. Through this research project we aimed to acquire data to assess the attitudes and perception of medical students towards flipped classroom at the Aga Khan University.

## METHODS

A group of second year undergraduate medical education (UGME) (n=98) students (age 18 to 22 year) was selected for this research, after obtaining their informed consent and ethical clearance from the institutional review board (4667-BBS-ERC-17). Six sessions of Year-II-UGME-Neurosciences module were allotted to be “flipped” during January till March 2017. The pre-session instructions and materials that consisted of video lectures were shared with the students via One 45 server at Aga Khan University (AKU). These pre-session lectures involved presenting information with visual graphics and real-life examples of the concepts being studied. In addition to video lectures, the students were also given specific reading materials pertinent to the concept being taught. Students in the flipped class were encouraged to complete the assigned tasks before coming to class. As it was difficult to track which students completed the pre-session tasks on time, they were asked to take a quiz or complete assigned homework and post it on an online server called Padlet. The two primary purposes of this were to more accurately record which students had completed the pre-session preparation prior to class, and to help motivate students to come prepared to the class. This pre-session activity was followed by a face-to-face in-class discussion with the facilitator present to help and address students’ queries. Students were given cases and related questions to solve either as individuals or in groups. Some group activities were also planned to enhance peer learning. We also implemented formative assessment to enhance active learning and motivate student engagement by using freely available software called ‘Kahoot’. At the end of the sessions a Likert scale survey was used to capture their perceptions of the learning experience. The survey was previously used and validated in a study.^8^

The data was analyzed in SPSS version 21(IBM, Chicago, IL, USA.). Frequencies were calculated and presented as percentages via graphical representation. Moreover, the mean score and standard deviation was calculated in order to identify positive and negative responses. Cronbach’s alpha was computed to measure consistency within the responses of students and the cut-off of 0.70 was set to declare good reliability. The following five-point scale was used: a score of 1 was a strong negative association towards the question, a 3 was neutral, and a 5 was a strong positive association towards the question. An open-ended question was also given at the end of questionnaire to get qualitative responses from the students’ about their experience of using FCR.

## RESULTS

**Fig.1:**
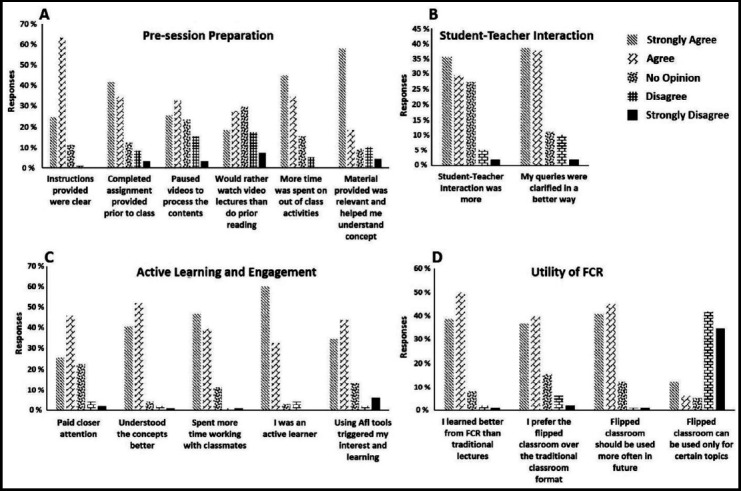
(A–D): Response to survey questions broadly classified into four domains. A) The replies of student regarding pre-session preparation are presented as percentages. Their responses regarding their studying strategy and clarity of instruction were noted. B) The overall feelings of students during the class session are presented. C) The feelings of the students regarding the FCR upon completion are presented. The students were very happy with the FCR sessions overall. D) Students’ opinion regarding the utility of FCR for future session is presented. Students were impressed with this mode of teaching and believed that it could be applied to more than particular topics only.

A total of ninety eight students participated in the survey with a male: female ratio of 68:30. Classification of the survey questions and their mean scores are shown below and frequencies in Fig.1 (A-D). The analysis of student responses for the open ended questions in flipped classes is shown in Table-I.

**Table-I:** Student Comments for the efficacy of Flipped Class Strategy.

*Themes*	*Student Comments*
Content Delivery	“The facilitators made sure to cover all concepts in simple terms and made too difficult concept easy to understand”
Video and Reading material	“Videos sometimes take a while to process, harder to take mental notes” “The studying bit before the class requires a little extra effort before the lecture” “Reading material should not be a preferred choice” “Funny learning video content made learning fun”
Session Scheduling	“Timing (8.30 am) was a little early for me to come prepared”
Unmotivated Students	“Some students did not come prepared, for the first session; but picked up the pace in later sessions”
Interaction driven learning	“I liked the concept of flipped classroom. It told us more about what we can expect from the class” “The fact that prior knowledge enabled interaction”
Formative assessment for learning	“Kahoot Qs related to pre-reading material i.e. formative assessment was the best part” “Quiz helped consolidate the concepts”

Some of the most common and unique comments of students regarding FCR are presented here. Students were in general happy with the FCR strategy and they also provided suggestions to improve future sessions.

### 1) Students perception of pre-session instructions/preparation

The minimum score was obtained for the item “I often paused the videos when watching them in order to process the content” (2.45 ± 1.13) while highest score recorded was for “The instructions provided for the non-face-to-face sessions were clear” (4.92 ± 0.67). Forty five % students were keener on watching the videos over completing reading tasks, and 75% of the class completed the preparation before coming to the assigned FCR session.

### 2) Student’s perception towards active learning and engagement

The maximum score of 4.83±0.75 was for the item “I actively participated in learning activities during FCR session”. This was evident by students’ engagement in activities as well as the enthusiasm shown by them towards formative assessment during the session (77%), though 8% students felt that these formative assessments were over whelming and demanded more time to be spent out of class by them.

### 3) Perception towards student-teacher interaction

A neutral but positive opinion was received regarding the student-teacher interaction domain of FCR from our students (3.63± 0.30) yet they felt that their queries and misconceptions were cleared in a much better way when compared to the traditional setting (4.09 ±1.04).

### 4) Utility of flipped classroom

The FCR model received high recognition and 84% of the students’ responded that it should be used more often and across the teaching years. Similarly, 87% said that they learned much better in FCR as compared to their regular classes.

## DISCUSSION

The results of our study show an overwhelming support for FCR as an effective mode to teach neuroscience. It was declared as better than the routine traditional lectures. Students believed that FCR was more engaging and fun and that this mode of teaching kept the classroom ‘alive’! These feelings were further reflected in the students’ agreeing that the FCR was a better learning experience and it should be routinely used for a diverse set of topics in medical curriculum. Flipped classroom teaching was found to be an effective method that improved medical students’ interest in learning and their self-learning abilities.^9^ Similarly, this model was preferred by participants’ of a flipped continued medical education (CME) classroom.^10^ As for the pre-session material provided the students suggested that they were more comfortable and preferred watching video lectures for non-face to face preparation rather than reading. This may be attributed to the fact that video lectures provide flexibility in learning and a chance to review and repeat the sessions.^11^ A vast majority felt that pre-class material given for preparation enhanced their lateral thinking and provided a better correlation between knowledge and application of the concept taught.

Furthermore, it’s commonly observed that students are easily distracted in classrooms, and the common culprit is either cell phones or laptops. To minimize student distraction, we implemented the use of freely available software called ‘Kahoot’ during FCR. This motivated the students to use these very same devices for enhanced active learning and motivated engagement, rather than distracting activities. Seventy seven percent of the class reported that they appreciated the use of formative assessments during the sessions. They commented that they were able to identify concepts that required further clarification.

Student’s learning atmosphere is a blend of physical, social, and psychological components. Implementing techniques that boost the learning environment in class room teaching enables progressive understanding of the topic especially in UGME setting.^12^ Similar to our findings, a study conducted at Ripah University, Pakistan using a similar pedagogy to teach third year MBBS student during clinical rotation, reported that students found FCR as a better mode of teaching in their setup as well.^7^ Although we are at an early stage of developing and adapting FCR, we are excited by the fact that our results are promising and we have the potential of progressing this approach to other features apart from teaching students, including evolving the healthcare system as done by other institutes.^13^,^14^ In addition, a research comparing the different modes of teaching reports that incorporating problem based learning, video lectures and other techniques proved to be an effective way of teaching theory and clinical skills in medical education.^15^,^16^ Most significantly, active learning experiences during the teaching learning sessions were highly preferred by our students, yet they shared their concerns regarding the under utility of this pedagogy in medical curriculum, that might help them in not only becoming active learners but attain optimal grades.

The FCR pedagogy was also found useful in teaching sensitive topics such as childhood disabilities and special education to undergraduate medical students^17^, for teaching specialized topics such as glaucoma and ocular trauma^18^, complex and multiple patient comorbidities in nursing practice^19^, ^20^, in learning electrocardiogram^9^, histology among medical students^21^ and even more so provides times to think critically.^22^

The facilitators of this study feel that one challenging aspect of the flipped classroom is providing ample material to students and generating thought provoking scenarios for the in-class sessions. Most instructors have limited experience creating or identifying the content and may fear the change might not be accepted in their setting or environment. However, capacity building through training sessions can cater for this issue.

## CONCLUSION

The findings of the study suggest that FCR teaching and learning pedagogy is an effective way of enhancing student engagement and learning. Thus, the newer educational technology can be an effective tool of teaching and learning in this rapidly changing technological world and be part of a comprehensive system for lifelong education.
